# Sex-Comparative Outcomes of the T-Branch Device for the Treatment of Complex Aortic Aneurysms

**DOI:** 10.3390/jcm12185811

**Published:** 2023-09-07

**Authors:** Petroula Nana, Katarzyna Jama, Tilo Kölbel, Konstantinos Spanos, Giuseppe Panuccio, Tomasz Jakimowicz, Fiona Rohlffs

**Affiliations:** 1 German Aortic Center, Department of Vascular Medicine, University Heart and Vascular Center, University Medical Center Eppendorf, 20251 Hamburg, Germany; tilokoelbel@googlemail.com (T.K.); spanos.kon@gmail.com (K.S.); giuseppe.panuccio@gmail.com (G.P.); f.rohlffs@uke.de (F.R.); 2 Department of General, Vascular and Transplant Surgery, Medical University of Warsaw, 02-006 Warsaw, Poland; katarzyna.jama@gmail.com (K.J.); tomek.jakimowicz@gmail.com (T.J.); 3 Vascular Surgery Department, University Hospital of Larissa, Faculty of Medicine, University of Thessaly, 41110 Larissa, Greece

**Keywords:** females, sex, mortality, major adverse events, spinal cord ischemia, branched repair

## Abstract

Introduction: Females are at increased risk of mortality compared to males after complex endovascular aortic repair. This study aims to examine sex-related peri-operative and follow-up outcomes in patients managed with the t-Branch device. Methods: A two-center retrospective analysis of patients managed with the off-the-shelf t-Branch device (Cook Medical Inc., Bjaeverskov, Denmark) between 1 January 2014 and 30 September 2020 was performed. Primary outcomes were sex-comparative 30-day mortality, major adverse events (MAEs) and spinal cord ischemia (SCI). Results: A total of 542 patients were included; 28.0% were females. Urgent repair and type I–III thoracoabdominal aneurysms were more common among females (52.6% vs. 34%, *p* = 0.01, and 57.1% vs. 35.8%, *p* = 0.004). Technical success was similar (97.4% vs. 96.9%, *p* = 0.755), as well as early mortality (16.2% in females vs. 10.8% in males; *p* = 0.084). SCI rates were similar between groups (13.6% vs. 9.2% *p* = 0.183). MAEs were more common in females; 33.7% vs. 21.4% (*p* = 0.022). Multivariate analysis did not identify sex as an independent predictor of adverse events. The 12-month survival rate was 75.7% (SE 0.045) for females and 84.1% (SE 0.026) for males (log rank, *p* = 0.10). Conclusions: Sex was not detected as an independent factor of mortality, MAEs and SCI within patients managed with the t-Branch device. Feasibility was high in both groups. No significant difference was shown in survival during the 12-month follow-up.

## 1. Introduction

Female sex has been characterized as a factor of increased mortality in patients managed for aortic aneurysms [1,2,3]. Mortality among female patients tends to be two to three times higher than among their male counterparts when being managed for infrarenal abdominal aortic aneurysms (AAA) or juxta-, para-renal or thoracoabdominal aneurysms (TAAAs) by either endovascular means or conventional open repair [1,3,4]. In terms of setting, female patients managed urgently are at higher risk for mortality at 30 days than those undergoing elective repair, while endovascular management seems to provide a survival benefit, when feasible [4,5]. Female anatomic factors, such as smaller access vessels, and device characteristics probably affect decision making in the application of endovascular repair in females [6]. However, advancements in devices’ profiles may increase the suitability of endovascular repair in the female population [7].

Similarly complex endovascular management is followed by significant differences in outcomes when sex differences are investigated [1]. Female patients have been shown to present higher 30-day and 12-month mortality compared to males following endovascular management with fenestrated (FEVAR) and branched (BEVAR) endovascular aortic repair [1,8]. Similar findings have been reported for open repair for TAAAs shows, with increased female mortality, especially in type II aneurysm [9,10]. No significant differences have been recorded between urgent and elective female patients in terms of outcomes, although early mortality and spinal cord ischemia (SCI) rates in female cohorts seem to represent an important concern [11].

Along these lines, the aim of this study was to examine the sex-related peri-operative and follow-up outcomes in patients managed with the t-Branch device for BEVAR.

## 2. Methods

### 2.1. Study Design and Patient Cohort

This is a two-center retrospective analysis of prospectively collected data of patients managed with BEVAR using the off-the-shelf t-Branch device (Cook Medical Inc., Bjaeverskov, Denmark) for degenerative or post-dissection TAAAs and para- and juxta-renal aneurysms. The STrengthening the Reporting of OBservational studies in Epidemiology (STROBE) statement was followed [12]. All patients were managed between 2014 and 2019, while data collection and follow-up outcomes continued up until 30 September 2020. The cohort was dichotomized into female and male groups. Urgent and elective cases were included. All patients managed for symptomatic or ruptured aneurysms, or cases managed for aneurysms of more than 90 mm, were characterized as urgent. Patients managed for acute aortic dissections were excluded from the analysis. The technical details of the repair strategy have been published previously [13]. The left axillary artery was the most common access for branches, while the percutaneous approach was chosen to establish femoral access [13].

### 2.2. Data Collection

Sex, age, aneurysm type (degenerative or post-dissection) and Crawford’s TAAA classification were recorded, as well as maximum aneurysm diameter. Comorbidities, including coronary artery disease (CAD), myocardial infarction (MI), previous coronary-aortic bypass (CABG) or coronary stent angioplasty (PTCA), hypertension (HT), dyslipidemia (DLP), tobacco use (ever or active), chronic obstructive pulmonary disease (COPD), diabetes mellitus (DM), chronic renal disease (CRD), cerebrovascular events (stroke; minor or major transient ischemic attack (TIA)) and peripheral arterial disease (PAD) were noted. The duration of operation, duration of fluoroscopy, volume of contrast, presence of endoleak at completion angiography and use of spinal drainage (prophylactic or therapeutic) were recorded.

Post-operative morbidity including acute kidney injury (AKI), myocardial infarction, stroke (major, minor or TIA), SCI (paraplegia or paraparesis), respiratory failure, ischemic colitis, and access complications were also collected. Peri-operative medical treatment information with respect to antithrombotic therapy (aspirin, clopidogrel, double antiplatelet therapy and anticoagulant medication) was also collected. The lengths of intensive care unit (ICU) and hospital stay (LOS) were both recorded and analyzed. Follow-up included clinical and imaging re-evaluation, with computed tomography angiography (CTA), at the 1st and 12th month, and yearly thereafter. Mortality and target vessel (TV) adverse events were recorded during follow-up.

All patients’ data were deidentified and inserted in a common local quality-improvement database. This study was performed in compliance with the Declaration of Helsinki, and no approval was required from the local ethics committee due to its retrospective design and unidentifiable information.

### 2.3. Definitions

Early mortality was defined as any death recorded within 30 days after the procedure. Technical success was defined as correct endograft deployment with TV patency without evident type I–III endoleak or limb occlusion at final angiography [14]. Any new-onset, immediate or delayed neurologic deficit of the lower limbs not attributable to other pathologic entities, including any paraplegia (classes 0–2 of the modified Tarlov’s Scoring Scale) or paraparesis (classes 3–5 of the modified Tarlov’s Scoring Scale) up to 30 days postoperatively was characterized as SCI [14]. Chronic renal disease (CRD) was characterized as any glomerular filtration rate (GFR) < 60 mL/min/1.73 m^2^ or need for dialysis, while AKI was the reduction of the baseline by >25% or any new-onset dialysis after repair [14,15]. Major adverse events (MAEs) at 30 days were considered the composite outcome of mortality, MI, respiratory failure requiring prolonged (>24 h longer than anticipated) mechanical ventilation or reintubation, renal function decline resulting in >50% reduction in baseline eGFR or new-onset dialysis, bowel ischemia requiring surgical resection or not resolving with medical therapy, major stroke, and paraplegia (grade 3), as specified by the latest reporting standards [14]. MAEs were analyzed on a per-patient basis. Myocardial infarction was defined as any acute coronary syndrome with typical clinical symptoms and/or electrocardiographic changes and/or troponin elevation. Meanwhile, access complication was defined as any local infection, hematoma or pseudoaneurysm needing interventional or surgical repair of the access site.

### 2.4. Outcomes

The primary outcomes of the analysis were sex-comparative 30-day mortality, MAEs (composite outcome, as defined by the reporting standards) and SCI rates in patients managed with t-Branch devices. The comparative survival during follow-up was assessed as secondary outcome.

### 2.5. Statistical Analysis

Continuous data are reported as mean ± standard deviation, while categorical data are expressed as absolute numbers and/or percentages. Independent two-sample t tests were used for normally distributed continuous variables, and the Wilcoxon rank sum test was used for non-normally distributed continuous and ordinal variables. Multivariable regression was performed using the Wald test to determine the independent association of risk factors with mortality, MAEs and SCI at 30 days, as well as to identify baseline differences between sexes. *p* values were considered significant when if <0.05. Kaplan–Meier estimates were performed to assess follow-up survival. Estimates were considered reliable in cases where standard error (SE) < 0.10. The log rank test was used to compare survival distributions. No adjustment for missing was performed. Statistical analysis was performed using SPSS 22.0 for Windows software (IBM Corp, Armonk, NY, USA).

## 3. Results

### 3.1. Total Cohort

In total, 542 patients were managed using the t-Branch device; 37.0% (203 patients) underwent urgent repair and 8.5% (46 patients) were ruptured aneurysms. Females represented 28.0% (154) of the cohort, while patients’ mean age was 70.5 ± 8.5 years. Regarding aneurysm extension, 498 (91.8%) patients were managed for TAAAs (6.1% type I, 13.5% type II, 22.5% type III, 44% type IV, and 5.7% type V). Technical success was 97.0% (526/542), while the total mortality rate was 12.3%. In total, 94 patients received a CSFD (17.3%). The SCI rate was 10.5%, and 4% was the paraplegia rate.

### 3.2. Baseline Characteristics

The female patients’ mean age was 71.0 ± 8.1, while male patients’ mean age was 70.4 ± 8.1 years (*p* = 0.42). Urgent repair rate was higher among female patients (52.6%, 81 females vs. 31.4%, 122 males, *p* = 0.01). Mean aneurysm diameter was similar between the groups. Type I–III TAAAs were more common among females: 57.8% vs. 35.8%, *p* = 0.004 (Table 1).

No differences were recorded in ASA scores between groups. Female patients presented worse estimated glomerular filtration rates (eGFR 59.8 ± 23.5 mL/min/m^2^ vs. 65.9 ± 23.4 mL/min/m^2^, *p* = 0.007) and lower body mass indexes (BMI 24.9 ± 4.4 kg/m^2^ vs. 26.5 ± 4.2 kg/m^2^, *p* = 0.001) compared to males. Previous EVAR was less common in females (5.2% vs. 18.0%, *p* < 0.001). The distribution of the remaining pre-operative characteristics between groups are presented in Table 2. A multivariate analysis confirmed that female patients exhibited lower eGFR (*p* = 0.003), BMI (*p* = 0.001) and previous EVAR (*p* = 0.001). However, the rate of urgent repair was independently related to female sex (*p* < 0.001).

### 3.3. Intra-Operative Details

Technical success was similar between groups: 97.4% among females and 96.9% among males (*p* = 0.76). Longer procedural and fluoroscopy times were recorded in females (258 ± 136 min vs. 235 ± 124 min, *p* = 0.04, and 66 ± 43 min vs. 56 ± 34 min, *p* = 0.01), while contrast volume use was similar between sexes (231 ± 79.5 mL vs. 224.8 ± 67.3 mL, *p* = 0.37). Six (3.9%) females required pre-operative debranching of the LSA vs. 11 males (2.8%, *p* = 0.53); all interventions were carotid–subclavian bypasses, except two left subclavian transpositions (one in each group). There was a similar rate of CSFD use between groups: 20.1% vs. 16.2% (*p* = 0.28). Distal landing to the infrarenal aorta was used in 19.6% of females vs. 12.6% of males (*p* = 0.038), while the remaining cases were all managed with landing to the iliac axes. The incidence of endoleak on completion of angiography was similar between the groups (*p* = 0.159; type I: 0.6% vs. 1.0%, *p* = 0.68; type II: 13.6% vs. 10.6%, *p* = 0.369).

### 3.4. Early Outcomes

Early mortality was 16.2% in females (25 patients) and 10.8% (42 patients) in males (*p* = 0.09). All patients died in-hospital within 30 days, except one patient who died after discharge (within the 30-day follow-up). The SCI rate was 13.6% among females and 9.2% among males (*p* = 0.18), while paraplegia was 4.5% vs. 3.8%, respectively (*p* = 0.72). MAEs, as defined by reporting standards, were more common in females, at 33.7% vs. 21.4% (*p* = 0.022), especially in the case of major stroke (1.3% vs. 0%, *p* = 0.011). AKI rates, eGFR and creatinine values at discharge were similar between the groups (*p* = 0.705, *p* = 0.40, and *p* = 0.42). All remaining adverse events are presented in Table 3. The ICU and LOS were similar between the groups. Multivariate analysis showed no differences in terms of mortality, MAEs and SCI on the basis of sex or repair setting (i.e., elective vs. urgent).

### 3.5. Follow-Up Findings

**Figure 1:**
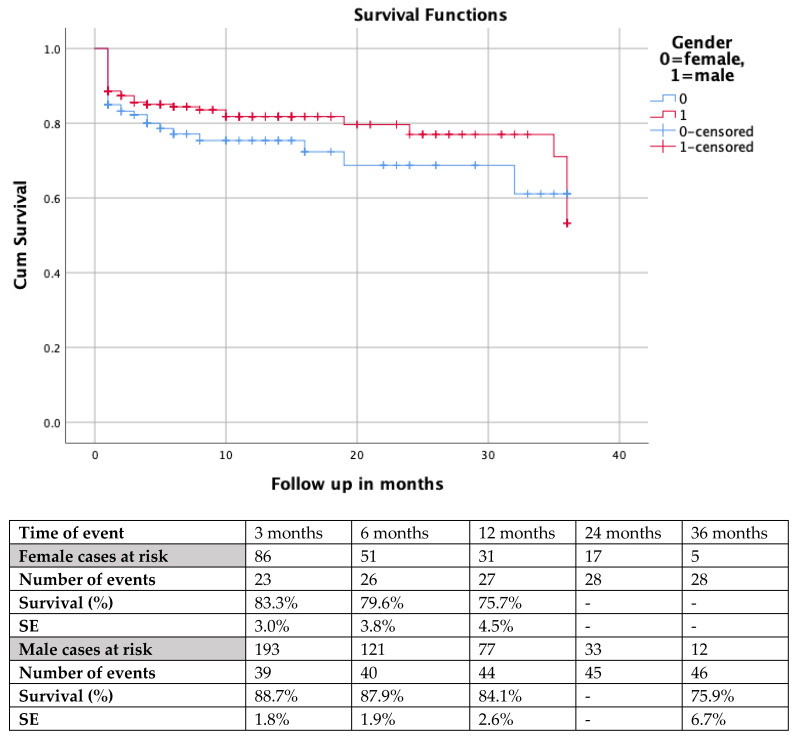
Kaplan–Meier scheme presenting follow-up mortality in females and males after complex endovascular repair using the t-Branch device. A non-significant difference was detected (log rank, *p* = 0.10). Footnotes: SE: standard error.

The mean follow up was 11.0 months (IQR 9; range 1–120 months) for the female cohort and 8 months (IQR 8; range 1–72 months) for males. The loss to follow-up was 49% for the female cohort and 53% for the male cohort. For females, survival rates were 83.8% (SE 0.03) at 3 months, 79.6% (SE 0.038) at 6 months and 75.7% (SE 0.045) at 12 months, while for the male cohort, the estimated survival was 88.7% (SE 0.018) at 3 months, 87.9% (SE 0.019) at 6 months and 84.1% (SE 0.026) at 12 months. No differences were detected in survival between the groups (log rank, *p* = 0.10). The Kaplan–Meier curves of comparative survival are presented in Figure 1.

Regarding TV patency, no differences were detected between the sexes for any type of TV. In total, eight events of TV occlusion were recorded in the female group and five in the male group, while all occlusions were detected within the initial 12 months of follow-up.

## 4. Discussion

Female patients are considered at higher peri-operative risk for death and/or adverse events compared to males when managed for aortic aneurysm repair, with either approach, endovascular or open repair and regardless of aneurysm extend; infra-renal or thoraco-abdominal aneurysms [3,16,17]. In this analysis, females presented similar outcomes to male patients. However, one in three females may be at risk to present any major complications during the early post-operative period. This finding did not seem to affect early mortality. Previous studies have shown that female sex has been detected as an independent factor of higher post-operative morbidity, including SCI, AKI, bowel ischemia and sepsis [16,17,18]. In the current study, when approaching MAEs separately, only major stroke rates were different between the groups; an outcome with controversial findings between sexes in the literature while neither complication related to access seemed to affect our findings [19,20].

Regarding the baseline characteristics, females presented higher rates of urgent repair and more extensive aneurysms; higher incidence of type I–III TAAAs (57 vs. 36%) while the eGFR at admission was lower in the female population [21,22]. The rate of CKD was similar between groups. Complex endovascular repair, including BEVAR, has been characterized as an effective and safe solution even in patients suffering with CKD; as it has not been shown to increase the risk of post-operative AKI rate or dialysis need in patients with declined renal function [22]. In addition, in the current cohort, the discharge eGFR was similar between sexes while for the female cohort, it increased compared to admission value. On the other hand, male patients presented higher cardiac comorbidity rates, including CAD, MI and CABG; a finding already described in the literature [23,24].

The main finding to have attracted interest in sex-specific analyses is mortality, which has been reported to be up to four times higher in the female population managed with FEVAR or BEVAR [1]. In this cohort, despite the fact that almost half of the female patients were managed urgently, 30-day mortality was similar between groups, as was survival at 12-month follow-up. It has already been shown that when excluding early mortality, survival at 12 months may not be affected by sex differences [1,21,25]. Postoperative morbidity related to technical failure, SCI and bowel ischemia may result in prolonged ICU and hospital stay, thus further increasing in-hospital mortality [25]. After the elimination of the peri-operative risks, survival up to 6 years seems to be similar between sexes [25]. The absence of difference in MAEs between sexes in this cohort, despite the higher MAE rate in females, may justify the similar early mortality rates between the sexes.

Technical success was similarly high between female and male patients, but females presented longer operation and fluoroscopy times, which is potentially related to their more complex anatomy. The current literature suggests that females may be at risk for lower technical success (86.2% vs. 96.6%) due to anatomy-related factors, such as more extensive aneurysms and smaller-caliber iliac access in almost one-third of patients, while female sex has also been identified as a factor of rotational deviation in patients managed with t-Branch devices [21,25,26]. Parameters including iliac access could potentially affect the findings of this analysis. However, due to missing data, an additional analysis on the impact of adjunctive procedures could not be performed. Despite the higher rate of type I–III TAAAs and the need for staged procedures in females, technical success was not affected. However, it should be acknowledged that distal landing above the aortic bifurcation was more common in the female cohort, indicating that additional technical adaptions may be needed to provide successful management in females, and an individualized approach, even when using off-the-shelf material, may assist in achieving favorable clinical and technical outcomes [25].

Despite a major stroke rate of 0.4% within the reported limits in the literature, a statistical difference was detected between females and males (1.3% vs. 0.0%, *p* = 0.011), potentially leading to a higher MAE rate [13,27]. Disease extension and need for proximal repair with TEVAR may affect the stroke rate in females [28]. Atherosclerotic burden of the aorta has been reported to be more significant than in males, while the extended operative and fluoroscopy time may depict additional wire or device manipulations within the aortic arch, which could result in higher embolic risk in the female cohort and explain the difference between sexes [29]. SCI rates were similar, potentially following the balance application of CSFD in both groups, as well the application of non-invasive measures for the prevention of SCI. Nevertheless, the estimated rate was 13.6% for females, probably as a result of extended aortic disease and the associated coverage; the urgent setting in the majority of cases should also be acknowledged [11].

### Limitations

The retrospective design and the sample size, including potential type-II statistical errors, may hamper firm conclusions, as they introduce significant risk of bias. The findings of this analysis represent the experience of two high-volume aortic centers and should be evaluated according to this parameter. Patients’ anatomical details, such as access vessel caliber, target vessel characteristics and the presence of thrombus, were not available in all patients, and could not be assessed, while specific information on the use of adjunctive procedure to establish access were not available or further assessed. Elective and urgent cases were included in this analysis, potentially affecting findings. The distribution of iliac landing using iliac branched devices was not evaluated and could potentially affect the outcomes of this cohort. Different types of aneurysms, such as thoracoabdominal and pararenal aneurysms, were managed, thus introducing additional bias, while at each involved center, prophylactic cerebrospinal drainage was applied using different protocols. This parameter could potentially have affected the SCI rate. The loss to follow-up should be acknowledged for both males and females, and all outcomes should be interpreted with caution in light of this parameter.

## 5. Conclusions

Sex was not detected as being an independent factor of mortality, MAEs or SCI in patients managed with the t-Branch device. Feasibility was high in both groups, despite that females presented higher TAAA I–III rate. MAEs were, however, more common among females. No significant differences were observed in survival at 12-month follow-up.

## Figures and Tables

**Table 1:** Distribution of pre-operative aneurysm characteristics between groups.

Aneurysm Characteristics	Female Cohort (154 Cases)	Male Cohort (388 Cases)	*p*
Aneurysm diameter (mm)	73.8 ± 5.9	76.8 ± 5.8	0.59
Thoracoabdominal aneurysm	143 (94.2%)	355 (91.5%)	0.83
I	21 (13.6%)	12 (3.1%)	<0.001
II	22 (14.3%)	51 (13.1%)	0.63
III	46 (29.8%)	76 (19.6%)	0.07
IV	44 (28.6%)	195 (50.3%)	0.008
V	10 (6.5%)	21 (5.4%)	0.65
I–III	89 (57.8%)	139 (35.8%)	0.004
Pararenal aneurysm	10 (6.4%)	4 (0.7%)	0.003
Juxtarenal aneurysm	1 (0.6%)	29 (7.5%)	0.002

**Table 2:** The distribution of comorbidities and ASA scores between female and male patients.

Comorbidities	Female Cohort (154 Cases)	Male Cohort (388 Cases)	*p*
Coronary artery disease	70 (45.5%)	217 (55.9%)	0.028
Myocardial infarction	21 (13.6%)	95 (24.5%)	0.005
CABG	8 (5.2%)	51 (13.1%)	0.007
PTCA/stenting	17 (11.0%)	30 (7.7%)	0.58
Hypertension	142 (92.2%)	355 (91.5%)	0.786
Dyslipidemia	89 (57.8%)	243 (62.6%)	0.279
Smoking	74 (48.1%)	218 (56.2%)	0.087
-Active smoking	38 (24.7%)	84 (21.6%)	0.447
COPD	30 (19.6%)	72 (18.6%)	0.778
Diabetes mellitus	24 (15.6%)	64 (16.5%)	0.795
Chronic renal disease	53 (34.6%)	166 (37.7%)	0.237
-Dialysis dependent	2 (1.3%)	7 (1.8%)	0.68
Stroke	12 (7.8%)	37 (9.5%)	0.52
Transient ischemic attack	3 (1.9%)	14 (3.6%)	0.32
Peripheral arterial disease	77 (50.3%)	39 (48.1%)	0.36
Previous aortic repair	45 (29.2%)	133 (34.3%)	0.26
Previous ascending repair	11 (7.1%)	14 (3.6%)	0.077
ASA score			
I	2 (1.3%)	9 (2.3%)	0.46
II	21 (13.8%)	76 (19.7%)	0.17
III	111 (73.0%)	261 (67.8%)	0.64
IV	18 (11.9%)	39 (10.2%)	0.62

ASA: American Society of Anesthesiology; CABG: coronary-aortic bypass; COPD: chronic obstructive pulmonary disease; PTCA: percutaneous coronary angioplasty/stenting.

**Table 3:** Early post-operative events, including 30-day mortality, spinal cord ischemia and MAE between the female and male group.

Post-Operative Events at 30 Days	Female Cohort	Male Cohort	*p*
Mortality	25 (16.2%)	42 (10.8%)	0.084
MAE	52 (33.7%)	83 (21.4%)	0.022
SCI	21 (13.6%)	36 (9.3%)	0.183
-Paraplegia	11 (7.1%)	15 (3.8%)	0.11
SIRS	7 (4.5%)	7 (1.8%)	0.07
Myocardial infarction	5 (3.2%)	5 (1.3%)	0.135
Respiratory failure	5 (3.2%)	7 (1.8%)	0.315
Stroke	5 (3.2%)	7 (1.8%)	0.315
-Major	2 (1.3%)	0 (0%)	0.011
Acute kidney injury	22 (14.3%)	50 (12.9%)	0.705
-Dialysis	7 (4.5%)	14 (3.6%)	0.624
Bowel ischemia	3 (1.9%)	7 (1.8%)	0.91
-Ischemia needing resection or not responding to medical treatment	1 (0.6%)	0 (0%)	0.14
Wound complications needing access site revision	33 (21.4%)	47 (12.1%)	<0.005

MAEs: major adverse events; SIRS: systemic inflammatory response syndrome; SCI: spinal cord ischemia.

## Data Availability

The data presented in this study are available on request from the corresponding author. The data are not publicly available as they represent anonymized hospitals databases.
